# Development and Validation of a Nomogram for Predicting Radiation-Induced Temporal Lobe Injury in Nasopharyngeal Carcinoma

**DOI:** 10.3389/fonc.2020.594494

**Published:** 2020-12-11

**Authors:** Wenqiang Guan, Kang Xie, Yixin Fan, Stefan Lin, Rui Huang, Qianlong Tang, Ailin Chen, Yanqiong Song, Jinyi Lang, Peng Zhang

**Affiliations:** ^1^ Department of Radiation Oncology, Sichuan Cancer Hospital & Institute, Sichuan Cancer Center, School of Medicine, University of Electronic Science and Technology of China, Radiation Oncology Key Laboratory of Sichuan Province, Chengdu, China; ^2^ Department of Oncology, The Second People’s Hospital of Yibin, Yibin, China; ^3^ The Second Department of Oncology, Chengdu First People’s Hospital, Chengdu, China; ^4^ Department of Computer Science and Engineering, Office for Student Affairs, School of Statistics, Economics Institute, University of Minnesota-Twin Cities, Minneapolis, MN, United States; ^5^ Viterbi School of Engineering Applied Data Science, University of Southern California, Los Angeles, CA, United States

**Keywords:** nasopharyngeal carcinoma, chemoradiotherapy, temporal lobe injury, neutrophil-to-lymphocyte ratio, nomogram

## Abstract

**Background:**

The purpose was to develop and validate a nomogram for prediction on radiation-induced temporal lobe injury (TLI) in patients with nasopharyngeal carcinoma (NPC).

**Methods:**

The prediction model was developed based on a primary cohort that consisted of 194 patients. The data was gathered from January 2008 to December 2010. Clinical factors associated with TLI and dose–volume histograms for 388 evaluable temporal lobes were analyzed. Multivariable logistic regression analysis was used to develop the predicting model, which was conducted by R software. The performance of the nomogram was assessed with calibration and discrimination. An external validation cohort contained 197 patients from January 2011 to December 2013.

**Results:**

Among the 391 patients, 77 patients had TLI. Prognostic factors contained in the nomogram were Dmax (the maximum point dose) of temporal lobe, D1cc (the maximum dose delivered to a volume of 1 ml), T stage, and neutrophil-to-lymphocyte ratios (NLRs). The Internal validation showed good discrimination, with a C-index of 0.847 [95%CI 0.800 to 0.893], and good calibration. Application of the nomogram in the external validation cohort still obtained good discrimination (C-index, 0.811 [95% CI, 0.751 to 0.870]) and acceptable calibration.

**Conclusions:**

This study developed and validated a nomogram, which may be conveniently applied for the individualized prediction of TLI.

## Introduction

NPC is characterized by unique geographic distribution and is particularly prevalent in East and Southeast Asia. Epidemiological trends over the past decade show that its morbidity has gradually decreased, and mortality has been greatly reduced (1). Concurrent chemoradiotherapy is the standard treatment for advanced NPC. Because the temporal lobe is adjacent to the nasopharynx anatomically, radiation-induced TLI is one of the most serious late complications after definitive chemoradiotherapy of NPC patients. In an era of Intensity Modulated Radiation Therapy (IMRT) radiotherapy, the reported rate of TLI ranges from 4.33 to 12.5% (2–5). Patients who developed temporal lobe necrosis after radiotherapy suffer damages in memory, language, mobility, and executive functions, yet their general intelligence remained relatively intact (6).

In recent years, some studies focused on identifying the risk factors leading to TLI (7–13). The accumulated dosage of radiation was generally considered with an important risk factor for TLI. Sun et al. reported that D0.5 cc was predicted for TLI in NPC patients (7). Zeng et al. and Kong et al. established NTCP for TLI including D1cc and Dmax (14, 15), but clinical utility is limited. Few studies have attempted to develop easily acceptable prognostic model, though some risk factors for TLI have been reported.

Generating user-friendly graphical interface is helpful to make clinical decisions by using nomograms during the clinic (16). So far, it has been published in many studies that nomograms were used to predict outcome (17–19). The main purpose of this study was to analyze the risk factors of TLI, develop a prognostic model, and validate it in an external cohort. To our knowledge, this is the first study to develop a TLI related nomogram.

## Materials and Methods

### Patients

The patients of this study were from January 2008 to December 2013 *via* tracking the institutional database for medical records. Included patients with histologically confirmed NPC underwent definitive concurrent chemoradiotherapy. The primary cohort was gathered from January 2008 to December 2010. A validation cohort contained patients who were from January 2011 to December 2013. The exclusion criteria of this study were as follows: 1) Recurrence patients; 2) The MR image of follow-up or radiotherapy plans were not retrievable from archived database; 3) Brain invasion. All patients relied on enhanced MRI to stage by using American Joint Committee on Cancer Staging Manual (8th Edition) staging criteria. Demographic, clinical, and treatment plan of the 391 eligible patients was collected.

### Treatment

The patients were fixed in a supine position with a thermoplastic mask. Treatment plan CT was finished after intravenous contrast, obtaining 3 mm slices from the head to the level 3 cm below the sternoclavicular joint. The primary nasopharyngeal lesions (GTVnx) and metastatic neck lymph nodes (GTVnd) were delineated based on the criterion of the International Commission on Radiation Units and Measurements (ICRU) 50 and 62 (20, 21). The clinical target volume 1 (CTV1) was defined as the GTVnx with 5 mm margins to cover the high-risk subclinical area. CTV2 was defined by addition of 3–5 mm margins for the CTV1 to encompass areas of the low-risk subclinical area. CTVln was defined as lymphatic drainage regions. The planning target volume (PTV) was defined by addition of 3 mm margins for the GTV and CTVs. The prescribed dose was defined as: 68–76 Gy for PTV of GTVnx, 66–70 Gy for PTV of GTVnd, 60–66 Gy for PTV of CTV1, 54–60 Gy for PTV of CTV2, and 50–54 Gy for PTV of CTVln. Total fractions were 30–33 times. The patients were irradiated once a day over 5 days per week. The dose–volume limitations for normal organs were based on the Radiation Therapy Oncology Group protocol 0225 (RTOG0225) (22). Concurrent chemotherapy included cisplatin-based chemotherapy every 3 weeks for 2 to 3 cycles.

### Diagnosis and Temporal Lobe Contour

**Figure 1:**
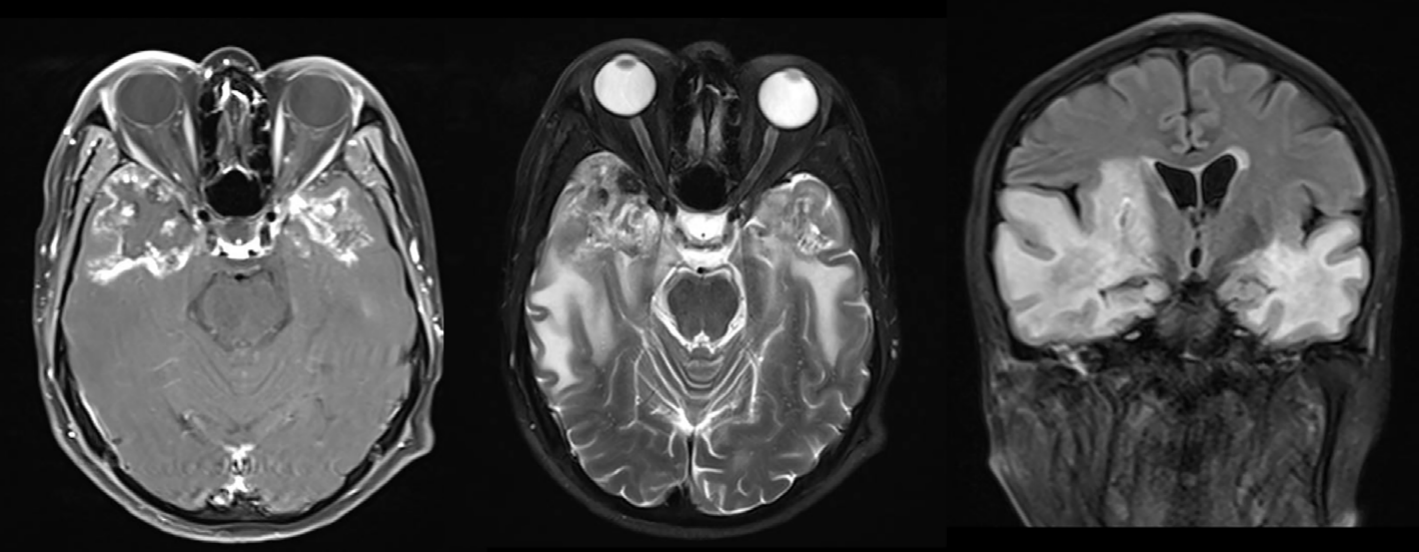
Typical MR images of radiation-induced temporal lobe injury (TLI).

The endpoint of this study was the development of TLI which was identified by enhanced MRI (**Figure 1**) after definitive concurrent chemoradiotherapy. The diagnostic methods were as follows: (a) white matter lesions, indicated finger-like pathological changes on T2-MRI with increased signal intensity; (b) contrast-enhanced lesions, defined as T1 enhancement scans showed abnormally spotted, circular, or irregularly enhanced lesions, with or without edema around the enhanced lesions; (c) cysts, extremely high signal strength on a T2-weighted image with round or oval shapes (23). All MR images were judged independently by two neuroradiology experts. As the temporal lobes had been delineated inconsistent during original radiotherapy planning, we re-contour the temporal lobes using a recommended atlas (24). This allows us to accurately collect data for the following dose–volume parameters: Dmax, Dmean, D1cc, D3cc, D5cc, D10cc, D15cc, and D20cc.

### Follow-up

The time of follow-up was computed from the completion of radiotherapy to either the day of last examination or the day of death. All patients who finished the radiotherapy were followed up every 3 months within 2 years and every 6 months within 5 years, then once a year thereafter. A detailed physical examination was done at each follow-up. Besides, MRI of the nasopharynx and neck, chest radiography, abdominal US were performed on every examination. The duration of TLI was calculated from the completion of radiotherapy to the day of contrast-enhanced MRI diagnosis.

### Development and Validation of a Nomogram

For patients with unilateral TLI, the uninjured temporal lobes were regarded as the normal temporal lobes for analysis. The statistical analysis of this study was based on 782 evaluable temporal lobes, which were divided into the injured temporal lobes and normal temporal lobes. The nomogram was developed based on parameter estimates of the multivariate logistic regression in the primary cohort. The equation was shown as below: *β* means the regression coefficient, X_1_, X_2_…Xm stand for different parameters.

$$\text{Probability of TLI }=\frac{e^{\left(\beta_{0}+\beta_{1}X_{1}+\beta_{2}X_{2}+\cdots +\beta_{m}X_{m}\right)}}{1+e^{-(\beta_{0}+\beta_{1}X_{1}+\beta_{2}X_{2}+\cdots +\beta_{m}X_{m})}}$$

For internal validation, the discriminative power of the nomogram was assessed by C-index, and the calibration was evaluated by the calibration plot. Bootstrap resampling (1,000 resamples) was used to calculate a relatively corrected C-index. For external validation, each patient was assessed and calculated by the nomogram in the validation cohort, and each patient’s total score was used by an independent factor of Logistic regression analysis. Then the discrimination and calibration for the nomogram were performed by the C-index and the calibration curve again.

### Statistical Analysis

The TLI and non-TLI groups were assessed by t test. The associations between clinical characteristics and the risk of TLI were evaluated using univariate logistic analysis. Multivariable analysis was performed using the logistic regression with forward stepwise selection, including all variables with P <0.05 on univariable analyses. Nomogram prediction model was developed by R soft. The Hosmer–Lemeshow test was used to evaluate the calibration curve.

All statistical analyses were carried out with SPSS version 25.0 or with R software (version 3.5.2; http://www.r-project.org). All tests were two-sided, P <0.05 was considered statistically significant.

## Results

### Clinical Characteristics

The median of the follow-up time was 42 months. 77 out of 391 patients developed TLI after definitive chemoradiotherapy. 665 normal temporal and lobes 117 injured temporal lobes were included in the statistical analysis. The median of the time period after the completion of radiation for the patients diagnosed with TLI was 36.5 months. Of the 77 patients with TLI, 37 patients were unilateral TLI, and the other 40 patients were bilateral TLI. There were 80 injured temporal lobes for bilateral TLI which were enrolled in this study. 782 evaluable temporal lobes’ characteristics in the primary and validation cohorts were given in **Table 1**. There was no significant difference between the two cohorts in TLI (P = 0.164). There were 39.4% more men than women. Most patients were diagnosed with advanced T stage (58.8%) and N stage (80%).

**Table 1:** Clinical characteristics of 782 temporal lobes.

Characteristics	All	Primary Cohort	Validation Cohort	*P*
	No. (%)	No. (%)	No. (%)	
**Total**	782	388	394	
**Sex**				0.071
**Male**	545(69.7%)	282(72.7%)	263(66.8%)	
**Female**	237(30.3%)	106(27.3%)	131(33.2%)	
**Age(years)**				0.005
**≤60**	688(88.0%)	354(91.2%)	334(84.8%)	
**>60**	94(12.0%)	34(8.8%)	60(15.2%)	
**T stage (AJCC8th)**				0.247
**T1**	36(4.6%)	18(4.6%)	18(4.65)	
**T2**	286(36.6%)	149(38.4%)	137(34.8%)	
**T3**	216(27.6%)	113(29.1%)	103(26.1%)	
**T4**	244(31.2%)	108(27.8%)	136(34.5%)	
**N stage (AJCC8th)**				0
** N0**	36(4.6%)	28(7.2%)	8(2%)	
**N1**	120(15.3%)	70(18%)	50(12.7%)	
**N2**	521(66.6%)	252(64.9%)	269(68.3%)	
**N3**	105(13.4%)	38(9.8%)	67(17%)	
**Hypertension**				0.256
**Yes**	48(6.1%)	20(5.2%)	28(7.1%)	
**No**	734(93.9%)	368(94.8%)	366(92.9%)	
**Diabetes**				0.742
**Yes**	31(4.0%)	15(3.8%)	16(4.1%)	
**No**	751(96.0%)	373(96.2%)	378(95.9%)	
**Smoking**				0.905
**Yes**	308(39.4%)	152(39.2%)	156(39.6%)	
**No**	474(60.6%)	236(60.8%)	238(60.4%)	
**Alcoholism**				0.007
**Yes**	216(27.6%)	124(32.0%)	92(23.4%)	
**No**	566(72.4%)	264(68.0%)	302(76.6%)	
**Cholesterol**				0.206
**≤5.2 mmol/L**	576(73.7%)	278(71.6%)	298(75.6%)	
**>5.2 mmol/L**	206(26.3%)	110(28.4%)	96(24.4%)	
**Triglycerides**				0.544
**≤1.7 mmol/L**	532(68.0%)	260(67.0%)	272(69.0%)	
**>1.7 mmol/L**	250(32.0%)	128(33.0%)	122(31.0%)	
**NLR**				0
**≤2.82**	456(58.3%)	202(52.1%)	254(64.5%)	
**>2.82**	326(41.7%)	186(47.9%)	140(35.5%)	
**PLR**				0.027
**≤117.53**	422(54.0%)	194(50%)	228(57.9%)	
**>117.53**	360(46%)	194(50%)	166(42.1%)	
**Target therapy**				0.774
**Yes**	134(17.1%)	68(17.5%)	66(16.8%)	
**No**	648(82.9%)	320(82.5%)	328(83.2%)	
**ART**				0.465
**Yes**	284(36.3%)	136(35.1%)	148(37.6%)	
**No**	498(63.7%)	252(64.9%)	246(62.4%)	
**TLI**				0.164
**Yes**	117(15%)	65(16.8%)	52(13.2%)	
**No**	665(85%)	323(83.2%)	342(86.8%)	

NLR, neutrophil-to-lymphocyte ratios; PLR, platelets-to-lymphocyte ratios; ART, adaptive radiation therapy; TLI, temporal lobe injury.

### Factors Associated With TLI

In the primary cohort, there were 65 injured temporal lobes, whose Dmax of temporal lobe were between 74.55 and 83.21 Gy, and D1cc was between 64.37 and 73.73 Gy. The other dose–volume parameters were shown in **Table 2**. The Dmax was the best dose–volume predictor with an AUC of 0.766. The dose of temporal lobe (Dmax, D1cc) in the TLI group was significantly higher than that of the non-TLI group (P < 0.05). For the clinical characteristics of primary cohorts, T stage, diabetes, and NLR were predictive factors with statistical difference (P < 0.05) (**Table 3**). However, only T stage (P <0.001) and Alcoholism (P = 0.031) were associated with TLI in the validation cohort.

**Table 2:** Dose–volume parameters of radiation temporal lobe injury in primary cohort.

Dose–volume parameters	TLI (mean ± SD Gy)	Non-TLI (mea ± SD Gy)	P	AUC
**Dmax**	78.88 ± 4.33	74.17 ± 4.77	0.000	0.766
**Dmean**	18.05 ± 7.14	16.15 ± 7.18	0.064	0.573
**D1cc**	69.05 ± 4.68	63.79 ± 8.53	0.000	0.691
**D3cc**	57.02 ± 7.57	54.68 ± 12.26	0.140	0.542
**D5cc**	49.46 ± 9.07	48.14 ± 13.68	0.456	0.516
**D10cc**	40.31 ± 10.93	37.18 ± 14.16	0.094	0.573
**D15cc**	33.03 ± 11.64	29.79 ± 13.54	0.074	0.578
**D20cc**	26.90 ± 11.34	24.41 ± 11.34	0.127	0.567

AUC, area under curve; SD, Standard Deviation.

**Table 3:** Univariate analysis of temporal lobes in primary and validation cohorts.

Characteristics	Primary cohort		*P*	Validation cohort		*P*
	TLI (+)	TLI (−)		TLI (+)	TLI (−)	
**Sex**			0.252			0.469
**Male**	51(78.5%)	231(71.5%)		37(71.2%)	226(66.1%)	
**Female**	14(21.5%)	92(28.5%)		15(28.8%)	116(33.9%)	
**Age(years)**			0.195			0.104
**≤60**	62(95.4%)	292(90.4%)		48(92.3%)	28(83.6%)	
**>60**	3(4.6%)	31(9.6%)		4(7.7%)	56(16.4%)	
**T stage (AJCC8th)**			<0.001			<0.001
**T1**	0(0%)	17(5.3%)		0(0%)	18(5.3%)	
**T2**	3(4.6%)	134(41.5%)		6(11.5%)	131(38.3%)	
**T3**	27(41.5%)	97(30%)		16(30.8%)	87(25.4%)	
**T4**	35(53.8%)	75(23.2%)		30(57.7%)	106(31%)	
**N stage (AJCC8th)**			0.175			0.092
**N0**	4(6.2%)	24(7.4%)		1(1.9%)	7(2%)	
**N1**	18(27.7%)	52(16.1%)		11(21.2%)	39(11.4%)	
**N2**	37(56.9%)	215(66.6%)		36(69.2%)	233(68.1%)	
**N3**	6(9.2%)	32(9.9%)		4(7.7%)	63(18.4%)	
**Hypertension**			0.69			0.118
**Yes**	4(6.2%)	16(5.0%)		1(1.9%)	27(7.9%)	
**No**	61(93.8%)	307(95.0%)		51(98.1%)	315(92.1%)	
**Diabetes**			0.035			0.402
**Yes**	6(9.2%)	9(2.8%)		1(1.9%)	15(4.4%)	
**No**	59(90.8%)	314(97.2%)		51(98.1%)	32(95.6%)	
**Smoking**			0.206			0.089
**Yes**	30(46.2%)	122(37.8%)		15(28.8%)	141(41.2%)	
**No**	35(53.8%)	201(62.2%)		37(71.2%)	201(58.8%)	
**Alcoholism**			0.947			0.031
**Yes**	21(32.3%)	103(31.9%)		6(11.5%)	86(25.1%)	
**No**	44(67.7%)	220(68.1%)		46(88.5%)	256(74.9%)	
**Cholesterol**			0.168			0.563
**≤5.2 mmol/L**	42(64.6%)	236(73.1%)		41(78.8%)	25(75.1%)	
**>5.2 mmol/L**	23(35.4%)	87(26.9%)		11(21.2%)	85(24.9%)	
**Triglycerides**			0.304			0.974
**≤1.7 mmol/L**	40(61.5%)	220(68.1%)		36(69.2%)	236(69%)	
**>1.7 mmol/L**	25(38.5%)	103(31.9%)		16(30.8%)	106(31%)	
**NLR**			0.003			0.273
**≤2.82**	23(35.4%)	179(55.4%)		30(57.7%)	22(65.5%)	
**>2.82**	42(64.6%)	144(44.6%)		22(42.3%)	118(34.55)	
**PLR**			0.892			0.565
**≤117.53**	32(49.2%)	16(50.2%)		32(61.5%)	196(57.3%)	
**>117.53**	33(50.8%)	16(49.8%)		20(38.5%)	146(42.7%)	
**Target therapy**			0.099			0.495
**Yes**	16(24.6%)	52(16.15)		7(13.5%)	59(17.3%)	
**No**	49(75.4%)	271(83.9%)		45(86.5%)	28(82.7%)	
**ART**			0.951			0.886
**Yes**	23(35.4%)	113(35.0%)		20(38.5%)	12(37.4%)	
**No**	42(64.6%)	210(65.0%)		32(61.5%)	21(62.6%)	

For the multivariate logistic regression analysis, only T stage, NLR, Dmax, D1cc were independent prognostic factors for TLI (**Table 4**). Among dose–volume parameter and clinical factors, Dmax(P = 0.033), D1cc (P < 0.001), advanced T stage (P < 0.001), and higher NLR (P = 0.012) were found to correlate with a higher incidence of TLI in primary cohort.

**Table 4:** Multivariate logistic regression analysis of TLI in primary cohort.

Variable	TLI		
	OR	95%CI	*P*
**T stage (T3–4 *VS* T1–2)**	14.69	4.41–48.86	<0.01
**NLR (>2.82 *VS* ≤2.82)**	2.22	1.19–4.16	0.012
**Dmax (>75 Gy *VS* ≤75 Gy)**	2.29	1.07–4.91	0.033
**D1cc (>67 Gy *VS* ≤67 Gy)**	4.25	1.90–9.54	<0.01
**Diabetes (Yes *VS* No)**	3.25	0.89–11.80	0.072

CI, confidence interval; OR, odd ratio.

### The Development of the Nomogram Model for TLI Prediction

**Figure 2:**
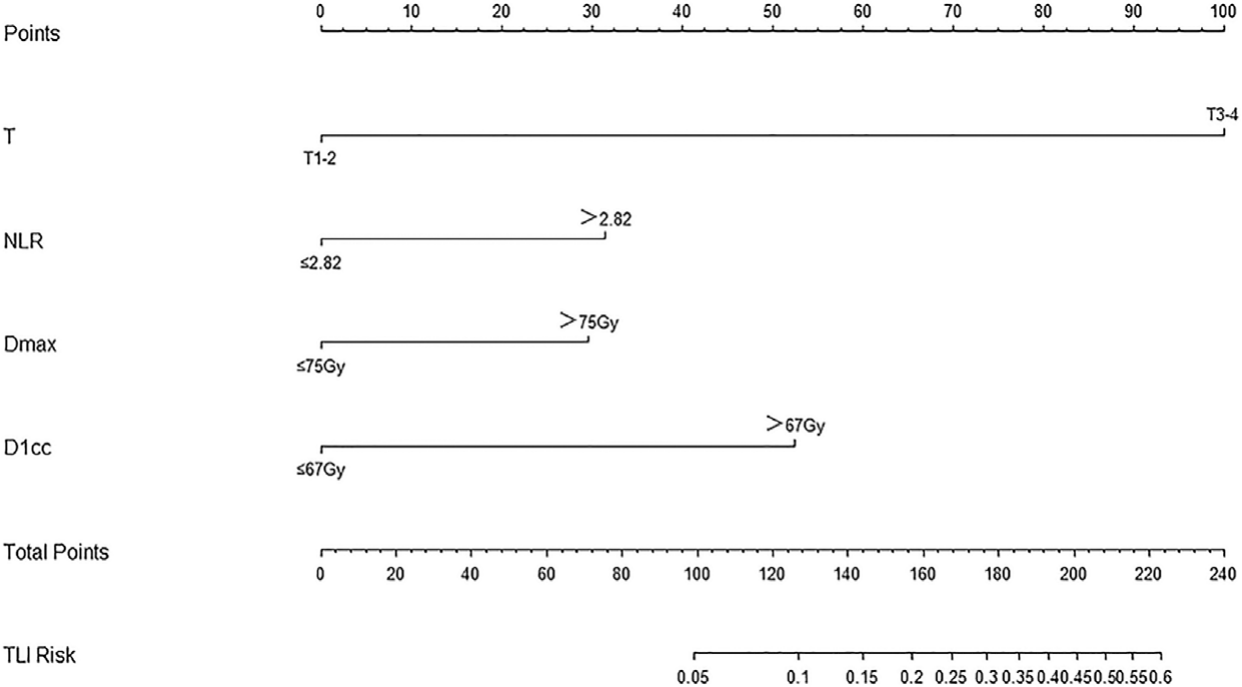
Nomogram for TLI risk after definitive concurrent chemoradiotherapy in nasopharyngeal carcinoma patients, including T stage, NLR, Dmax, and D1cc.

The nomogram for TLI was developed by R (**Figure 2**). The model’s parameters were from the results of the multivariate logistic regression analysis. The different states of each factor correspond to specific scores on the score scale. The individual’s calculated total score that takes into account all factors of the model can easily predict TLI risk by positioning it on the total score scale.

### The Discrimination and Calibration Ability of the Nomogram

#### Internal Validation

**Figure 3:**
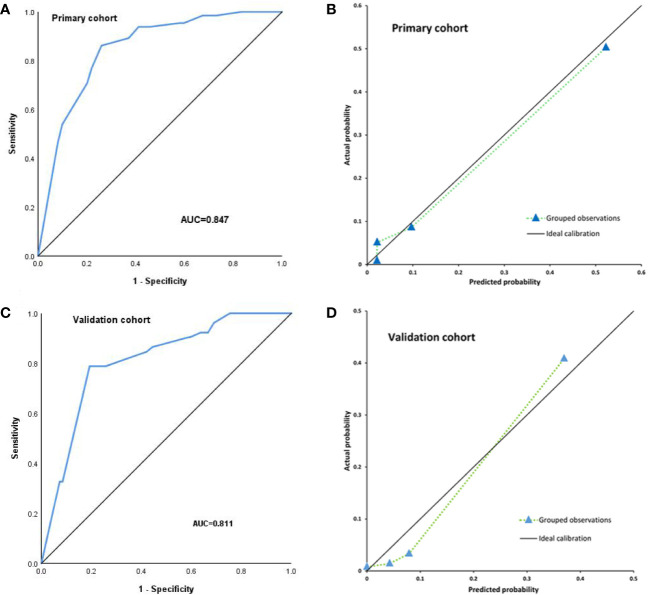
Internal validation for TLI risk in the primary cohort by nomogram; the area under the receiver operating characteristic curve (AUC) was 0.847 **(A)**. The calibration curve for the prediction of TLI risk in the primary cohort **(B)**. External validation for TLI risk in the validation cohort by nomogram, the area under the receiver operating characteristic curve (AUC) was 0.811 **(C)**. The calibration curve for the prediction of TLI risk in the validation cohort **(D)**.

The C-index was used to assess the discrimination of nomogram model (**Figure 3A**). The nomogram showed good discrimination power for predicting TLI with a C-index 0.847(95%CI, 0.800–0.893), which was subjected to be 0.841 *via* bootstrapping resampling. In **Figure 3B**, the nomogram demonstrated good calibration according to the Hosmer–Leme show test (P = 0.24). The x-axis represents the predicted probabilities from the nomogram, and the y-axis means the observed TLI probabilities. The calibration curve also indicated satisfactory consistency between the probability of prediction and observation in the primary cohort.

#### External Validation

For external validation, the nomogram was also evaluated by the calibration plot and the C-index in an independent validation cohort. The C-index of the nomogram for the prediction of TLI risk was 0.811(95%CI, 0.751–0.870) in the external validation (**Figure 3C**), which showed that the model has a good discrimination. The calibration plot indicated that the prediction model was well calibrated, and the TLI risk demonstrated an acceptable agreement between the actual observation and prediction results of the nomogram (P = 0.053) (**Figure 3D**).

## Discussion

TLI is a severe adverse event associated with definitive concurrent chemoradiotherapy of NPC that presents a slow progressive course. According to MRI, the white matter lesions (WMLs), contrast-enhanced lesions and cysts were continuous process. The WML was observed in all radiation-induced brain injury, while incidence of cysts was less than one-fifth of cases. Enhanced lesions and cysts always occurred with WML (23). Wang J et al. (25) carried out a similar study that the prediction model consisted of the dose of temporal lobe (D0.5 cc and d10 cc), the parameter selection of the model and developing came from LASSO regression. The differences in parameter selection and statistical methods may result in slightly different results. To our knowledge, this is the first study to develop a TLI related nomogram based on the clinical and dose–volume parameters. It can provide a visible predictive model that was easily understood by physicians and patients. When clinician reviewed the radiotherapy plan, they may individually adjust the radiation dose based on the nomogram for TLI. For example, local radiotherapy boost of nasopharyngeal carcinoma should be warned for including all risk factors. The nomogram was developed by R software. Internal validation showed that the subjected C-index was 0.841 according to Bootstrap resampling (1,000 resamples). The calibration curve also showed satisfactory consistency between the probability of prediction and observation. Applying the model to the external validation, the C-index of the nomogram for the prediction of TLI risk was 0.811.

The temporal lobe is located in the middle cranial fossa adjacent to the cavernous sinus and rupture hole. NPC can invade structures such as ruptured holes and cavernous sinuses through the anatomic space of the skull base and may even invade the temporal lobe. When irradiating tumors and subclinical lesions, radiation could be given a higher dose in the target area. The temporal lobe is inevitably exposed to higher doses of radiation during treatment. So advanced T stage may easily develop to TLI. Huang et al. reported the cumulative incidence of TLI at 5years was 13.2% among T4 NPC patients (3), Su et al. also showed TLI is not observed in T1–2 patients; the incidences are 3.1 and 13.4% in T3 and T4 patients respectively (8). For our study, the incidence of TLI seemed higher than previously reported in related research. Firstly, in order to ensure the integrity and reliability of the data, we have strict exclusion criteria, such as the MR images of follow-up or radiotherapy plans were not retrievable from archived database. Secondly, most patients presented with T3–4 stage (58.8%). However, it would not affect the performance of the model. James CH Chow et al. (26) evaluated radiation-induced hypoglossal nerve palsy in nasopharyngeal carcinoma. 797 patients were included after further excluding patients whose treatment plans were not retrievable from archived database; dose–volume data from 165 eligible patients were analyzed to develop a model for predicting radiation hypoglossal nerve injury.

The precise mechanism that leads to TLI remains unknown; it may be related to vascular damage (27). TLI was likely to be related with the volume and dose of temporal lobe irradiated. Zeng et al. found Dmax to the temporal lobe was a significant factor affecting TLI (11). Su et al. reported NPC patients who received definitive concurrent chemoradiotherapy were relatively safe with Dmax <68 Gy or D1cc < 58 Gy in temporal lobe (8). In this study, we noticed that Dmax and D1cc were independent prognostic factors for TLI in multivariate logistic regression. So Dmax and D1cc were associated with developing TLI and incorporated into the nomogram model.

Many studies have shown that the ratio of neutrophils to lymphocytes in the blood can be used to predict the outcome of various cancers and inflammatory diseases (28, 29). Wu et al. found the associations of blood circulating neutrophil-to-lymphocyte ratios (NLR) with TLN occurrence in T4 NPC patients (30). Similarly, we got the same result that NLR was an independent prognostic factor to result in TLI. It suggested that inflammatory factors played roles in the late brain damage caused by concurrent chemoradiotherapy.

A study limitation was that data is retrospective firstly. But the included patients’ data was complete and has detailed follow-up records; the quality of the data was relatively reliable. Secondly, the MRI diagnosis of TLI was not fully established, but we have assigned two neuroradiologists to examine each MRI independently.

## Conclusions

We have developed and validated a nomogram for TLI in an independent cohort. Additional research is needed to evaluate whether this nomogram can be applied to other populations.

## Data Availability Statement

The raw data supporting the conclusions of this article will be made available by the authors, without undue reservation.

## Ethics Statement

The studies involving human participants were reviewed and approved by the Department of Radiation Oncology, Sichuan Cancer Hospital & Institute, Sichuan Cancer Center, School of Medicine, University of Electronic Science and Technology of China, Radiation Oncology Key Laboratory of Sichuan Province. Written informed consent for participation was not required for this study in accordance with the national legislation and the institutional requirements.

## Author Contributions

WG, KX, and PZ: conceived and designed this study. WG, KX, YF, RH, YS, QT, and AC: material preparation and data collection. WG and JL: statistical analysis. JL and PZ: study review and monitoring. WG and KX: visualization, manuscript writing and submission. All authors contributed to the article and approved the submitted version.

## Conflict of Interest

The authors declare that the research was conducted in the absence of any commercial or financial relationships that could be construed as a potential conflict of interest.
